# Cross-Dimensional Mapping of Number, Length and Brightness by Preschool Children

**DOI:** 10.1371/journal.pone.0035530

**Published:** 2012-04-19

**Authors:** Maria Dolores de Hevia, Monica Vanderslice, Elizabeth S. Spelke

**Affiliations:** 1 Université Paris Descartes, Sorbonne Paris Cité, Laboratoire Psychologie de la Perception, CNRS UMR 8158, Paris, France; 2 Department of Psychology, Harvard University, Cambridge, Massachusetts, United States of America; National Institute of Mental Health, United States of America

## Abstract

Human adults in diverse cultures, children, infants, and non-human primates relate number to space, but it is not clear whether this ability reflects a specific and privileged number-space mapping. To investigate this possibility, we tested preschool children in matching tasks where the dimensions of number and length were mapped both to one another and to a third dimension, brightness. Children detected variation on all three dimensions, and they reliably performed mappings between number and length, and partially between brightness and length, but not between number and brightness. Moreover, children showed reliably better mapping of number onto the dimension of length than onto the dimension of brightness. These findings suggest that number establishes a privileged mapping with the dimension of length, and that other dimensions, including brightness, can be mapped onto length, although less efficiently. Children's adeptness at number-length mappings suggests that these two dimensions are intuitively related by the end of the preschool years.

## Introduction

Number and space appear to be intimately related in the human mind. Recognition of this relationship already was evident from the early description of ‘number forms’, which consist of spontaneously generated mental images where each number occupies a constant position in a spatial configuration, usually a line [1]. During the last decades, an increasing body of evidence has shown that when adults engage in numerical processing, and even when numbers are irrelevant to the task at hand, their performance in behavioral tasks such as comparison, line bisection, or stimulus detection, exhibits spatially-related effects that are consistent with the hypothesis of a spontaneous mapping of numbers onto an oriented space [2]–[5], as well as onto a non-oriented space related to spatial extent [6]–[8]. The number-space link is further suggested by neuroimaging studies, which reveal partially overlapping regions of parietal cortex activated by tasks tapping numerical and spatial cognition [9], and by studies showing that neural circuits dedicated to eye movements are recruited during arithmetic, providing evidence for spatial shifts of attention during performance of numerical addition [10]. Finally, neglect patients who are unable to attend to the contralesional side of space have been shown to present deficits in numerical tasks that tap onto an oriented spatial representation of number [11], [12]. Nevertheless, all the subjects in the above tasks were educated and living in a culture that makes the number-space mapping prominent through measurement devices, use of number lines in schools, scales on maps, and the like. Thus, the number-space mapping may partly reflect culture-specific experience instead of a predisposition of the human mind to connect these two dimensions.

What accounts for this relation? On one view, the mapping of number and space is specific and privileged [13]. On a second view, the mapping of number and space results from two general properties of the human mind: a propensity to encode all continuous dimensions in a common framework, and a propensity to map any dimension of variation to any other [14]–[16]. On a third view, space is a privileged dimension of experience, against which all other dimensions are measured and compared [17], [18]. We consider each of these views in turn.

For children at the start of formal schooling, the mapping of number to space is revealed on tasks in which children are asked to place symbolic numbers on a line segment bounded by two numbers. At all ages tested, children show monotonically increasing placements, suggesting that they readily map numbers to horizontal spatial positions, although children's placements change from logarithmic to linear with increasing age [19]–[21]. Studies of uneducated adults living in a remote Amazonian community, and tested with non-symbolic numerical displays (arrays of dots), provide evidence that abilities to construct number lines are universal, and that education, rather than age, accounts for the shift from logarithmic to linear placement of non-symbolic numbers [22]. Moreover, it has been recently shown that the association between non-symbolic number and space is present in preschool children at 5 years of age [23] and it traces back to infants, who generalize from an increasing or decreasing sequence of non-symbolic numbers to an increasing or decreasing sequence of spatial lengths, and who show learning and generalization of a rule that establishes a positive, but not an inverse, relationship between number and length [24]. The mapping is also present in non-human primates: the intraparietal sulcus of macaques has been shown to not only contain intermingled populations of neurons that respond to discrete quantity (arrays of forms varying in number), and to continuous quantity (spatial length), but even a subset of neurons that are tuned to both numerosity and length [25]. Nevertheless, none of these experiments has revealed whether the mapping of number to length is specific to these dimensions or is more general.

Some evidence suggests that the number-space mappings shown by non-human animals, infants, preschool children, and adults in remote cultures reflect both the existence of a generalized magnitude system [16], and the ability to map any dimension of variation to any other [26]. Comparisons of numbers and lengths are governed by the same psychophysical function, Weber's law, whereby discrimination performance is modulated by the ratio of the magnitudes rather than by their absolute difference [27], [28]. But Weber's law applies to a variety of continuous dimensions for adults, including line length [27], size of named animals, objects or countries [29], [30], and abstract magnitudes like the intelligence or ferocity of animals [31]. Weber's Law also governs infants' discrimination along many dimensions such as number, duration and size, describing an interesting developmental profile: large ratios are required for the youngest infants [32], smaller ratios for infants aged 6 months [33]–[36], and still smaller ratios at 9 months of age [34], [37]. This common signature suggests that the different dimensions of magnitude are characterized by a common comparison function [38].

However, the few studies directly addressing the hypothesis of a single system of magnitude offer conflicting evidence. In Stroop-like tasks, some authors report interference effects at both the behavioral and anatomical levels between symbolic number and size, and between size and brightness, but not between number and brightness [13], while others report mutual interference effects between symbolic number, size and brightness [39]. It has also been suggested that developmental changes in comparison abilities reflect changes in a domain-general comparison process [40], while other studies suggest that numerical and non-numerical comparison tasks modulate distinct brain regions [41]. In fact, although mappings between any continuous dimensions are plausible via their shared structural similarity [42], both a functional and a neural overlap could explain why some dimensions, like time, space and number share a special link [15], [24], [43], [44]. It is thus an open question whether any continuous dimension can be readily mapped onto any other early in development, and whether mappings between all types of magnitudes have equal status.

A third view is that space is a privileged medium for representing all other magnitudes. This view was articulated most clearly by Shepard [17], [45], [46], whose research showed that humans tend to represent variation along any dimensions in terms of spatial distance. Consistent with this view, some evidence suggests that space is a special medium for representing different continuous dimensions including time [43], color [18], [47], faces [48], the pitch of sounds [49], [50], geometric forms [46], discrete categories such as letters and months [51], [52], and elements in serial learning [53]. Thus, children may be predisposed to form mappings that give spatial content to any experienced dimensions. Nevertheless, adults and infants have been found to relate one spatial dimension –length– more readily to the duration than to the loudness of an accompanying sound [44].

In summary, the wealth of evidence for number-space mappings is consistent with different accounts of the source of these mappings. The present research was undertaken to test the different possibilities through studies of dimensional mappings in preschool children. We investigated and compared mappings among three dimensions –non-symbolic number, length, and brightness– in children aged 3.5 to 5 years. We chose as a third, non-spatial, dimension, the brightness contrast of figures, since this is the dimension that has received most attention in previous studies [13], [39], [40], and because it allows us to test all the dimensions with stimuli in the same format.

We presented children with a series of matching tasks whereby the numbers of forms in a visual array, the lengths of individually presented lines, and the brightness levels of single visual forms were related systematically to one another. If the common nature of these dimensions, as continua, allows for comparable, interchangeable mappings between them, then children should show equal accuracy in all of the matching conditions: number to length, number to brightness, and brightness to length. If all mappings of space to other dimensions are privileged, then children should show high and equal abilities to map length to number and to brightness but be less able to map number to brightness. If the number-length mapping is privileged, then children should perform best when mapping across these two dimensions.

We tested these contrasting predictions in two steps. First, we designed a mapping task and investigated whether children could spontaneously discriminate each of the dimensions of brightness, length, and number as they were instantiated in our displays, and use the dimensions to perform the matching task (Experiment 1). Children were found to succeed at the task with all three dimensions, setting the stage for the critical tests of children's mapping across these dimensions (Experiment 2). To this end, we tested children on all six positive mappings between number, length, and brightness (e.g., from increasing brightness to increasing length), as well as on three of the six inverse mappings (e.g., from increasing brightness to decreasing length). By comparing positive mappings in the two orders (e.g., from number to length vs. length to number), we investigated whether children's mappings across dimensions are symmetric. By comparing children's performance with positive vs. inverse mappings, we investigated whether there is a specific direction to each of the mappings, as has been observed for the number-length mapping in infants [24].

## Results

### Experiment 1: Intra-dimensional mappings of number, length, and brightness

**Figure 1:**
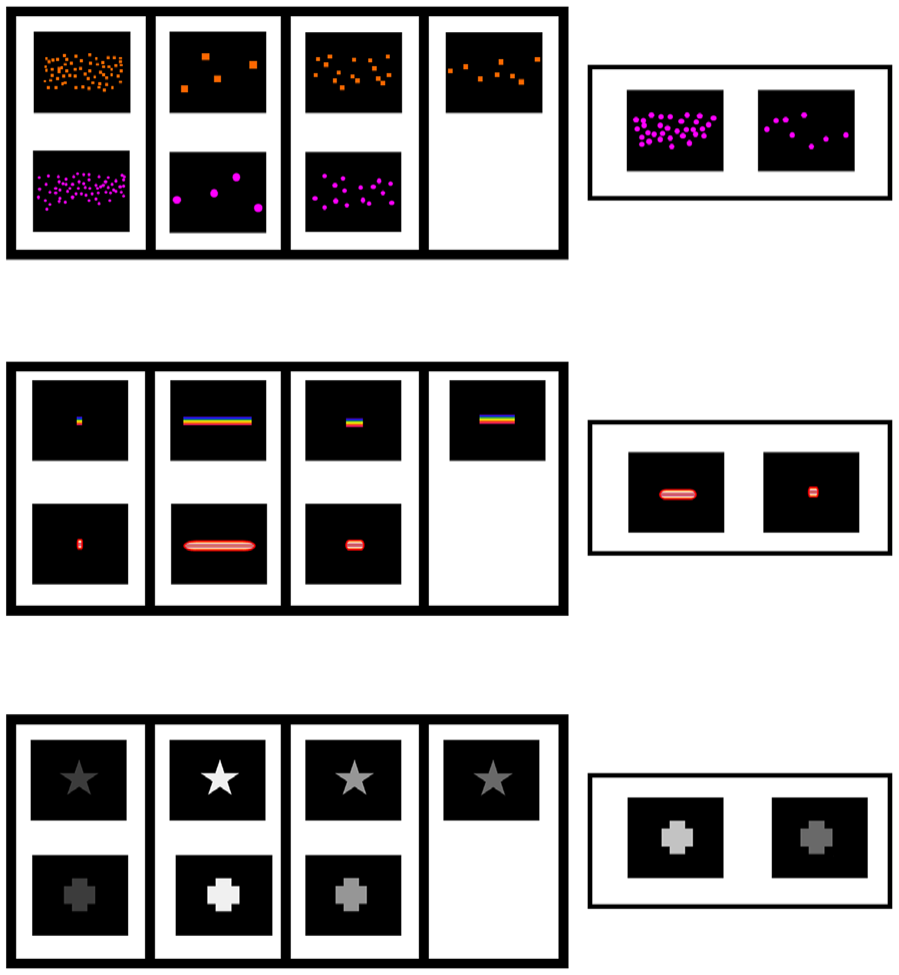
Examples of stimuli for the intra-dimensional mappings. (top) Numbers are matched to equal numbers; (center) line lengths are matched to equal line lengths; and (bottom) levels of brightness are matched to equal levels of brightness.

The first experiment tested children's abilities to form mappings within each of the dimensions of number, length, and brightness (Figure 1). Children were asked to play a matching game using a set of cards depicting different numbers of objects, lines of different lengths, and figures with different levels of brightness. The game consisted of matching two sets of four cards with the same number of objects, with lines of equal length, or with figures of the same level of brightness, over changes in the particular objects depicted on the cards (for example, from a card depicting four triangles to one depicting four squares, or from a card depicting a bright cross to one depicting a bright star). On each trial, the experimenter presented the child with one set of four cards depicting variation on the chosen dimension, and then performed the first three matches on that dimension by placing three cards in a second set next to the corresponding cards in the first set. Finally, the experimenter pointed to the fourth card in the first set and showed the child two new test cards, one of which matched the standard on the chosen dimension. The child was asked to perform the last match by choosing between these two test cards.

Children performed each of the mappings reliably, with highest accuracy in the brightness condition (86%, chance = 50%, t_23_ = 8.57, p<.0001), followed by the length condition (79%, t_23_ = 5.06, p<.0001) and the number condition (65%, t_23_ = 2.37, p = .02). A one-way ANOVA comparing accuracy across the three conditions showed a significant effect (F_2,69_ = 4.15, p = .02). LSD post hoc tests revealed significantly greater accuracy for brightness than for number (p = .006) and marginally greater accuracy for length than for number (p = .063). This difference in accuracy did not result from a speed-accuracy tradeoff, as there was no significant difference in RT between the mappings (F_2,46_ = 2.19, p = .12), and the average RT for a trial in the brightness mapping was actually the fastest (3.5 s), relative to the length (4.2 s) and the number (4.6 s) mappings. Three separate one-way ANOVAS comparing accuracy for each dimension across females and males showed no significant sex effects for either the brightness and length conditions, (both Fs<1, n.s.), or for the number condition (F_1,22_ = 1.76, p = .2).

Thus, children understood the matching task and were able to perform it successfully for all three tested dimensions. Indeed, children performed significantly better for the brightness matching than for the numerical matching. An advantage for comparisons across the dimensions of height and brightness relative to numerical stimuli has been previously reported in both children and adults in studies where magnitude changes across the three dimensions were performed similarly to the present study [54]. The present findings show that the matching task is meaningful to children and that the differences in magnitude for the three dimensions were successfully discriminated, at least for the cards used in the test trials. Because brightness is detected as well as, or better than, number and length in the present displays, any failure to map number or length to brightness could not plausibly be attributed to lack of discrimination of the test cards depicting different levels of brightness. Experiment 2 therefore used the present task to investigate children's mappings across the three dimensions of number-length, number-brightness, and length-brightness.

### Experiment 2: Inter-dimensional mappings between number, length, and brightness

**Figure 2:**
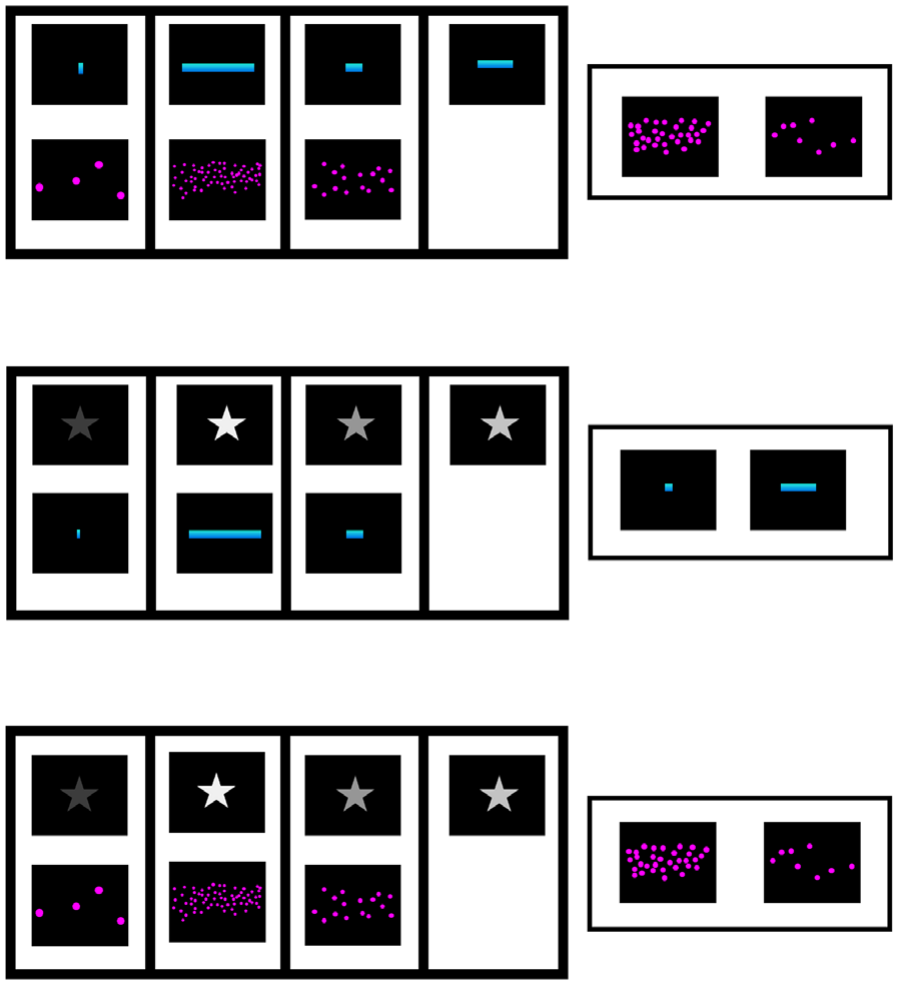
Examples of stimuli for inter-dimensional mappings. (top) a positive mapping between numbers and length; (center) a positive mapping between brightness and length; (bottom) a positive mapping between numbers and brightness.

The task presented in Experiment 2 used the same stimuli as in Experiment 1 but paired differently, such that mappings were performed inter-dimensionally (Figure 2). These inter-dimensional mappings did not require children to produce exact equivalents for each instance of the dimensions, as classical experiments with adults on cross-dimensional matching [55], [56], but to match the stimuli according to the monotonic increase or decrease on each perceived dimension. We tested children across the six possible positive mappings and three of the six possible inverse mappings (length to number, length to brightness, and brightness to number).

For the positive mappings between number and length, cards with numerosities were matched to cards with line lengths, such that the larger the number the longer the line, or cards with line lengths were matched to cards with numerosities, such that the longer the line the larger the number. In the inverse mapping, cards with line lengths were matched to cards with numerosities, such that the longer the line the smaller the number.

For the positive mappings between brightness and spatial length, cards with levels of brightness were matched with cards with line lengths, such that the brighter the figure the longer the line, or cards with line lengths were matched to cards with levels of brightness, such that the longer the line the brighter the figure. In the inverse mapping, cards with line lengths were matched to cards with levels of brightness, such that the longer the line the darker the figure.

For the positive mappings between number and brightness, cards with numerosities were matched to cards with levels of brightness, such that the larger the number the brighter the figure, or cards with levels of brightness were matched to cards with numerosities, such that the brighter the figure the larger the number. In the inverse mapping, cards with levels of brightness were matched to cards with numerosities, such that the brighter the figure the smaller the number.

Because mapping across dimensions may be an unusual task for children, each child was given three practice trials with intra-dimensional mappings (number-number, brightness-brightness, length-length), followed by three test blocks of inter-dimensional mapping, one with each pair of dimensions (number and length, number and brightness, and length and brightness). No training or feedback was given for the inter-dimensional mapping trials. Two separate groups of children were tested with each positive mapping in each direction (e.g., from number to length vs. from length to number). A third group of children was tested with inverse mappings of the three pairs of dimensions.

**Figure 3:**
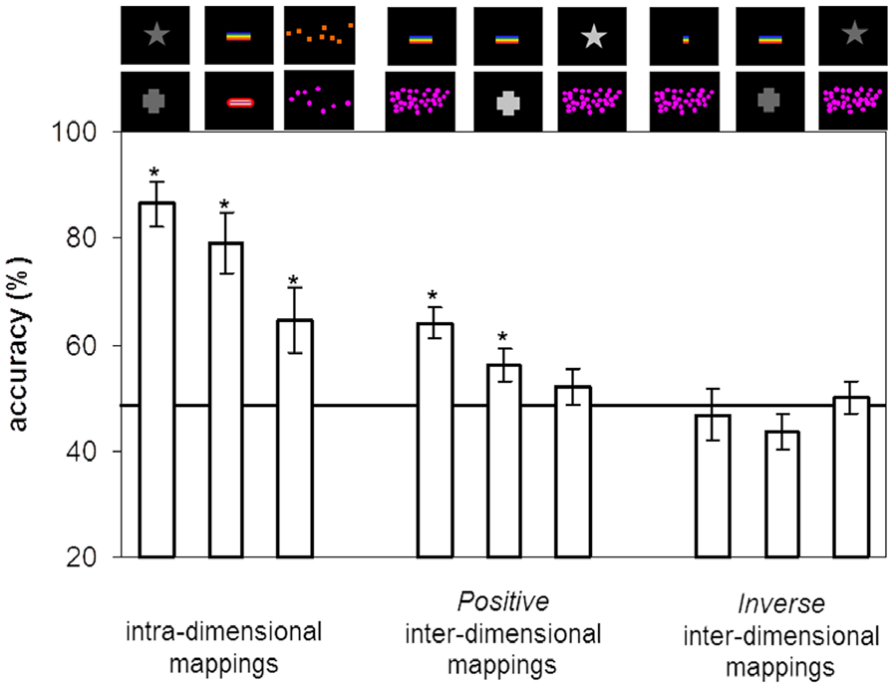
Overall mean accuracy (%) for the intra-dimensional mappings of brightness, length, and number, and the inter-dimensional mappings of length/number, length/brightness, and brightness/number, in both positive and inverse directions. The horizontal bar represents accuracy at chance (50%). The asterisks above the bars denote that performance was significantly above chance.

For the first positive mapping group, performance (%) was compared to chance level (50%). Children performed significantly above chance for the mapping of number to length (t_23_ = 3.14, p = .0004), while for the mappings of number to brightness and brightness to length performance was not different from chance level (both t_23_<1, n.s.). For the second positive mapping group, both the mappings of length to number (t_23_ = 3.71, p = .001) and of length to brightness (t_23_ = 2.14, p = .04) were significantly above chance, but not the brightness to number mapping (t_23_<1, n.s.). Performance from the two groups of children receiving positive mappings did not significantly differ as a function of the direction of the mapping: number-to-length (63%) vs. length-to-number (66%); brightness-to-length (54%) vs. length-to-brightness (58%); number-to-brightness (55%) vs. brightness-to-number (49%) (all three independent-samples t-tests: t_46_<1, n.s.). Collapsing data across the two directions of each positive mapping, performance exceeded chance (50%) on the mappings between both number and length (64%, t_47_ = 4.89, p = .00001), and length and brightness (56%, t_47_ = 2.00, p = .05). In contrast, performance was not different from chance for the number-brightness mappings (52%, t_47_<1, n.s.) (Figure 3).

Performance for the three inverse mappings did not significantly differ from chance level (50%; all three ps>.08). In order to test whether there was a privileged direction on the mapping we compared performance from the positive mappings to the inverse ones. Performance for the positive mappings of number and brightness did not significantly differ from the performance for the inverse mapping (52% vs. 50%, independent-samples t-test: t_70_<1, n.s.). In contrast, performance was significantly better for the positive than for the inverse mappings between number and length (64% vs. 47%, independent-samples t-test: t_70_ = 3.24, p = .001), and between brightness and length (56% vs. 44%, independent-samples t-test: t_70_ = 2.48, p = .01). Thus, children were not equally able to learn an equally predictable rule that establishes an inverse relationship between dimensions of length and number, and length and brightness: children associated longer lines more reliably to brighter objects and larger numbers than to darker objects and smaller numbers.

In order to investigate whether the mapping between number and length had a special status among the other inter-dimensional mappings, we conducted a one-way ANOVA for the positive mappings (number and length, brightness and length, number and brightness). Performance accuracy was indeed significantly different as a function of the mapping (F_2,141_ = 3.76, p = .02). LSD post hoc tests showed that accuracy was significantly higher for the number and length mapping than for the number and brightness mapping (p = .007). In contrast, accuracy on the number-length mappings was not significantly higher than accuracy on the brightness-length mappings (p = .08). Performance on the mappings of brightness and length, and brightness and number, did not differ (p = .3). Reaction times did not differ across the three conditions, (F_2,94_<1, n.s.). Moreover, the reliable difference in accuracy across the positive number-length and number-brightness mappings did not result from a speed-accuracy tradeoff, because the mappings of number and length were performed at least as fast (3.99 s) as the mappings of number and brightness (4.34 s).

Binomial tests showed a significant difference between the numbers of subjects performing better at each of the positive number-length vs. positive number-brightness mappings (both ps = .02), and no other significant differences (all other ps>.2). This significant difference was confirmed when collapsing across the two positive mapping conditions (p = .03), with the other contrasts still being not significantly different (all ps>.2).

**Figure 4:**
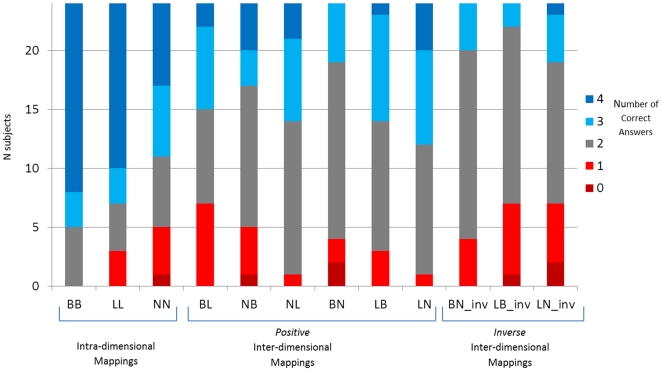
Distribution of correct answers (0, 1, 2, 3, 4) across participants for each of the mappings (intra-dimensional, two positive inter-dimensional, and inverse inter-dimensional). Each vertical bar represents the number of participants (total N = 24). Statistical analyses showed a significant difference between the red (0 and 1 correct responses) and the blue (3 and 4 correct responses) zones for all the intra-dimensional mappings, for all the positive number-length mappings, and one positive length-brightness mapping.

Further binomial tests focused on the numbers of children in each condition who performed well (3 or 4 correct answers) or poorly (0 or 1 correct answers) for each of the intra-dimensional and inter-dimensional mappings. For all the intra-dimensional mappings, the numbers of children performing well was significantly higher than the number of children performing poorly (all ps≤.01). For the inter-dimensional mappings, however, only the positive length-brightness (p = .02) and the two positive mappings between number and length (both ps<.01) had a significantly higher number of children performing well (Figure 4).

An ANOVA with Mapping (brightness-number, length-brightness, length-number) x Direction (positive, inverse), focusing on the positive and inverse mappings where both the referent and the variant dimensions were the same, showed a significant main effect of Direction (F_1,138_ = 11.09, p = .001) and a significant interaction between Mapping and Direction (F_2,138_ = 3.47, p = .03). LSD post hoc tests showed that the difference between the positive and inverse mappings was significant for the length-brightness (p = .01) and for the length-number (p = .001) mappings, but not for the brightness-number mappings (p = .08). These results suggest that the mappings of number and length, and brightness and length have a privileged direction, relative to the mappings of number and brightness.

Why did children fail to map number to brightness? In order to solve the inter-dimensional mappings, children needed to represent number, length and brightness as continuous, oriented dimensions of magnitude. Failure to do so, however, cannot explain children's failure on the number-brightness mapping task. Children successfully discriminated the magnitudes represented in the test trials across the three dimensions in Experiment 1: indeed, they performed best on the brightness task. Moreover, children successfully mapped increasing number and increasing brightness to increasing, but not decreasing, length. Thus, children represented the brightness dimension, as well as the number and length dimensions, as oriented from lesser to greater magnitude. Children's differing performance across the three mapping conditions therefore provides evidence against the general magnitude representation view, and supports the third view: children were able to learn a rule that established a positive relationship between space and two other continuous dimensions: number and brightness.

Although performance on the two mappings involving length did not differ significantly from each other, several features of the data suggest that children are especially adept at mapping length to number. First, for inter-dimensional trials involving number, children were reliably better at performing mappings of this dimension with length than with brightness. Moreover, both groups receiving positive mappings of number and length performed significantly above chance level, while only one group receiving positive mappings of brightness and length performed reliably above chance. Finally, number was the most difficult dimension for mapping in Experiment 1, but children nevertheless showed a consistently high performance for number-length mappings in Experiment 2. Moreover, performance for the intra-dimensional mapping of number was not significantly better than either of the two positive inter-dimensional mappings of number and length (unpaired t-tests, both ts<1, n.s.). Therefore, mapping a number to a similar number was about as difficult for children as mapping a number to a corresponding line length. These findings suggest that the hypothesis of a privileged mapping of number to space should not be ruled out, even though the findings of Experiment 2 do not decisively confirm it.

## Discussion

This study provides evidence that preschool children perform mappings across some perceptual dimensions more readily than others. In particular, children performed reliably above chance in mappings between number and length, and partially between brightness and length, but not between number and brightness. The finding that positive mappings involving length were successful support the view that spatial length is a privileged dimension onto which other dimensions are readily mapped, including number and brightness. Moreover, children's consistently higher performance mapping between number and length, relative to mapping between number and brightness, offers some support for the view that number and space are closely related for children, prior to the onset of formal schooling.

This study also shows that the mapping between number and length, and brightness and length, has a specific direction: children succeeded with a positive mapping between these dimensions but failed when they were related through an inverse rule. The finding accords with evidence from pre-verbal infants, who are able to learn and generalize a rule that establishes a positive, but not an inverse, relationship between number and length [24]. In contrast, children failed at mappings between number and brightness for both the positive and inverse mappings. Other studies have reported a successful mapping between these dimensions [42]; however, the stimuli in our task did not include higher-order relationships between the dimensions, such as symmetry (e.g., oOo) and monotonicity (e.g., ooO), which act as Gestalt grouping principles, and might have helped children to successfully solve the mappings in previous studies.

This study reveals that mappings between different continuous dimensions do not have the same status, at least for preschool children. The results therefore do not support the view of a general ability to create equally meaningful mappings between any perceptible, oriented dimensions, and accord with previous reports testing patterns of interference between the same three dimensions in adults [13]. In the present study, when the dimension of number had to be mapped to other dimensions a reliable better performance for mappings with the dimension of length than with brightness was observed. Nevertheless, the present research only tested mappings across the dimensions of number, length, and brightness: although children created successful mappings involving length and other dimensions, it is possible that they are equally adept at mappings across other pairs of dimensions. Considerable research suggests that humans and animals are predisposed to map number to time [38], [57], and space both to time [43], [44] and to tonal relations [58]. Research using the present methods might fruitfully compare children's sensitivity to these different mappings.

Number and length show a functional and neural overlap that might explain why mappings between these two dimensions are so prominent. However, we cannot exclude that the consistently high performance for number-length mappings was partially due to cultural factors that emphasize relations between numbers and points in a line, for instance. In fact, the association between numbers and spatial positions is partially modulated by reading/writing habits [2], [59], and is even malleable through changes in context [60], [61]. Note, however, that the mapping between number and length tested in this study is a non-directional one that is apparent from early infancy to adulthood [7], [15], [24], while the orientation of the mental number line has been described later in development [62]–[64]. Also, the fact that in the numerical displays perimeter was correlated with number might have helped to form a stronger representation of magnitude. It is unlikely, however, that the mapping was based on the perimeter variable: other studies have revealed that for the large number range, as was used in our study, number is the most salient attribute of the arrays even if other non-numerical continuous variables are present in the visual displays [65], [66].

The present research adds to the evidence that relations between number and length are meaningful also for preschool children in a task that measures the spontaneous ability to create mappings across different dimensions. This relation, however, might depend on the status of space as a preferred dimension onto which other dimensions are mapped. Although studies with primates suggest that relations between number and space derive from an intrinsic cognitive architecture that links these dimensions, possibly traceable to common, evolutionarily ancient origins [25], these studies did not test the presence of a link between space and continuous dimensions other than number. The origins and development of inter-dimensional mappings, and their breadth and limits, therefore provide rich terrain for future research.

## Materials and Methods

### Experiment 1: Intra-dimensional mappings of number, length, and brightness

#### Participants

Twenty-four children (10 female, mean age 47.7 months, range 41 months to 59 months) participated in this experiment. Children were recruited from the Boston area and were tested in the lab or day care after a parent gave written informed consent.

#### Ethics Statement

The experiment was conducted after obtaining Institutional Review Board approval from the Department of Psychology at Harvard University. All participants' parents gave informed written consent before testing began.

#### Materials

Figure 1 presents the stimulus materials for this study. Stimuli consisted of sets of 10 cm by 8.5 cm cards depicting different numbers of objects, line lengths, or levels of brightness, on a black background. Differences on magnitude across the three dimensions were varied in such a way that ensured that changes were highly discriminable. The cards had Velcro on the back, to allow them to be affixed to a game board (62 cm×29 cm) with the available space for placing two rows of four cards each.

For the number trials, cards depicted 4, 8, 16, 32, or 64 colored circles, triangles, or squares. On each trial, the experimenter performed the matches for the numerosities 4, 16, and 64, while the child matched a card of either 8 or 32 elements to a second card presenting one of those numbers in an array of different objects. To encourage children to base their matches on number rather than on item size or summed area, summed area was equated for the numerosities 4, 16, and 64 by varying item size inversely to number, and item size was equated for numerosities 8 and 32. Thus, neither spatial cue could be used to perform the correct match: during the matches performed by the experimenter total area could not serve as a cue, while during the match made by the participant item size was uninformative. The space occupied by the elements was equated across all numerosities, and single elements were randomly positioned inside that area, varying across trials.

For the length trials, cards depicted horizontal lines formed by colored rectangles, ovals, or twisted curves at constant height and at five different lengths varying on the same 2∶1 ratio used for numbers: 4, 8, 16, 32, 64 mm. On each trial, the experimenter performed the matches for the lengths 4, 16 and 64 mm, while the child matched a card of either 8 or 32 mm to a second card presenting one of those lengths. To encourage children to focus on length rather than shape or global appearance, the two sets of cards presented figures that differed in color and form. For the brightness trials, cards depicted crosses, smiling faces, or stars, presented at 5 levels of brightness against a black background, so that the contrast between the figure and the background produced for adults marked changes in perceived brightness. Stimuli were created by manipulating the brightness percentage of the same figure at equivalent steps from 20% brightness (since 0% equals black) to 100% brightness, which corresponds to white. The brightest display both had the highest luminance and the greatest brightness contrast, since the latter has been found to determine the psychological direction of the continuum: the larger the contrast is associated to the larger the number [67]. On each trial, the experimenter performed the matches for the cards of brightness 20%, 60%, and 100%, while the child matched a card of either 40% or 80% brightness to a second card presenting one of those brightness levels. To discourage global matching and encourage a focus on brightness contrast, the two sets of cards presented different objects. The brightness contrast was the same across different lengths and numbers, and the size and number of objects were constant across different brightness displays. Thus, all three types of trials required children to perform a match on one dimension (number, length, or brightness) while ignoring variation in other dimensions (color and form for the number and length trials; form and object kind for the brightness trials).

#### Design

Children were presented with 3 blocks of four trials each, one block per dimension (number, length and brightness). Block order was counterbalanced across participants. The order of the first three matches performed by the experimenter was pseudorandom, so that consecutive changes in magnitude did not follow any predictable order. Because matches performed by the experimenter included the cards with numerosities 4, 16, and 64, the ratio between consecutive matches was either 1∶4 or 1∶16. For the fourth match, the two possible magnitudes were presented twice per block in a pseudorandom order, such that the ratio between the third and the fourth match was always 1∶2. The position of the two choice cards was counterbalanced across trials, so that each magnitude appeared twice in each location. The correct choice appeared therefore twice on each side of the board. Since the experimenter performed the matches using the cards that included the smallest, largest, and one of the medium values, children were calibrated on the range of possible values of magnitude across the three dimensions prior to the test trials.

#### Procedure

Children were seated at a small table in front of the experimenter and asked to participate in a matching card game. For each trial, the experimenter placed one card on the first row of the board saying ‘this one’, and then placed another card in the same position of the second row saying ‘matches this one’. The experimenter followed this procedure for the first three matches, following a left-to-right orientation with respect to the participant. For the fourth match, the experimenter placed the first card saying ‘and for this one I need your help’, and consecutively placed in front of the participant a little board with two affixed cards, horizontally arranged, asking ‘which one of these you think that matches this one?’, while pointing to the last card placed on the board. The participant either placed one of the cards on the board or pointed to it, in which case he/she was encouraged to place the card in the appropriate spot on the board. Since we were investigating the ability to spontaneously match cards based on the magnitude variable, children always received positive feedback (e.g., ‘good job; let's do another match’). Both accuracy and reaction times were coded. Reaction times were measured starting at the moment the board was placed in front of the participant until she/he took or pointed to one of the cards.

### Experiment 2: Inter-dimensional mappings between number, length, and brightness

The methods were the same as in Experiment 1, except as follows.

#### Participants

Twenty-four children (14 female, mean age 49 months, range 43 months to 58 months) participated in the first positive mapping condition receiving the positive number-to-length, length-to-brightness, and number-to-brightness mappings. Twenty-four children (12 female, mean age 51 months, range 44 months to 59 months) participated in the second positive mapping condition receiving the positive length-to-number, brightness-to-length, and brightness-to-number mappings. Twenty-four children (12 female, mean age 52 months, range 45 months to 60 months) participated in the inverse mapping condition receiving the inverse length-to-number, brightness-to-length, and brightness-to-number mappings.

#### Design and Procedure

Children on each group were first given three intra-dimensional matching trials on each dimension (number, line and brightness). During this practice phase, no informative feedback was provided for children participating in the first positive mapping condition, and 19 additional children were eliminated from that condition because they failed to correctly perform the task during the practice phase. In order to reduce attrition, children participating in the second positive condition and in the inverse condition received informative feedback on their performance for these intra-dimension practice trials. After one failure, the experimenter said ‘look, this one matches this one’, pointing to the correct match; then the experimenter presented a new trial within the same dimension. All children succeeded on this new trial. After the training phase, each group of children (the two positive groups and the inverse group) received four test trials for each of the inter-dimensional mappings. No informative feedback was provided for the inter-dimension test trials.

Children's accuracy was computed for each dimensional mapping (% correct performance across the four trials). Two positive mappings (e.g., from number to length vs. from length to number) were used in order to explore whether exchanging the referent and the variant stimuli [68] would affect mapping performance. Each of these mappings was compared to chance performance. Then the two positive mappings were compared to each other in order to test whether both directions of the mapping (e.g., from number to length vs. from length to number) were processed similarly. Next, each positive mapping was compared to the corresponding inverse mapping to test whether there was a privileged direction to the mapping across the two dimensions. Finally, an analysis of variance compared performance on the three types of positive mappings: number-length, brightness-length, and number-brightness.
